# Evaluating the impact of rehabilitation nursing intervention on quality of life in patients with burn injuries: A protocol for systematic review and meta-analysis

**DOI:** 10.1097/MD.0000000000023879

**Published:** 2021-01-08

**Authors:** Jun Xiang, Qing Yang, Wei-Guo Xie, Jing Zhou, Xiang Gong, Wei-Dong Zhang, Hong Wu

**Affiliations:** Department of Burns, Tongren Hospital of Wuhan University & Wuhan Third Hospital, Wuhan, Hubei, P. R. China.

**Keywords:** burns, injury, quality of life, rehabilitation nursing, systematic review

## Abstract

**Background:**

Despite the availability of pharmacological intervention, patients with burn injuries experience pain during the treatment of wounds. Supplementary rehabilitation nursing intervention are required to enhance the wellbeing of patients sustaining injuries from burns. The present study aims to conduct a systematic exploration of the impact of rehabilitation nursing intervention on the wellbeing in patients sustaining burn injuries.

**Methods:**

The electronic databases listed below will be searched systematically: PubMed, EMBASE, CINAHL, Cochrane Library, Web of Science, Scopus, China National Knowledge Infrastructure, and WanFang database. All the databases will be searched from their inauguration to November 2020. There will be no language constraints. Independent undertaking by 2 authors will select studies, extract data from selected studies, and assess the quality of the included studies. All disagreements will be resolved through discussion, or by consulting a third independent author. This study will make use of RevMan 5.3 software to perform statistical analysis.

**Results:**

The present protocol summarizes high-quality evidence to assess the impact of rehabilitation nursing intervention on the wellbeing of patients sustaining burn injuries.

**Conclusion:**

The results of the present protocol has the potential to present evidence to assess whether rehabilitation nursing intervention can enhance the wellbeing of patients sustaining burn injuries.

**Registration number:**

November 17, 2020.osf.io/t6b8c/. (https://osf.io/t6b8c/).

## 1 Introduction

Burns are injuries that are caused to the skin and tissues through heat. Burns are primarily caused by heat; however, they are also caused by radioactive radiation, electric shocks, friction, or contacting strong chemicals (eg, acids).^[1]^ All across the world, injuries from burns are considered as a serious health issue. In 2004, there were nearly 11 million cases where the incidence of burns were severe enough to necessitate medical attention.^[2]^ Moreover, among, burns are the fourth main reason for hospitalization of children with injuries.^[3]^ Injuries from burns can result in morbidity, extended periods of hospitalization, and disability.^[4–8]^ Burn care primarily aims to realize survival before restoring function and cosmesis. Throughout treatment, pain management and any psychological impacts are also considered.^[9]^ Comprehensive rehabilitation nursing encourages patients with burns to recover through rehabilitation training, psychological intervention, and discharge guidance. It is necessary to elevate the quality of life and overall wellbeing of patients sustaining injuries from burns; however, there is a lack of evidence-based medical research. Therefore, the present study conducts a systematic and comprehensive evaluation of the impact of rehabilitation nursing intervention on the wellbeing of patients sustaining burn injuries.

## 2 Methods

### 2.1 Study registration

This study is registered on the Open Science Framework (OSF, http://osf.io/). The registration number is DOI 10.17605/OSF.IO/T6B8C. It will be reported in accordance with the Preferred Reporting Items for Systematic Review and Meta-Analysis Protocols (PRISMA-P) statement.^[10]^

### 2.2 Eligibility criteria

#### 2.2.1 Types of studies

The present study will include all high-quality prospective cohort studies and all available randomized controlled trials (RCTs) assessing rehabilitation nursing intervention on the life standard of patients having injuries from burns. Case reports, reviews, conferences, comments, and studies involving animals are excluded.

#### 2.2.2 Types of participants

Regardless of age, gender, race, and country, this study includes all participants with burn injuries.

#### 2.2.3 Types of interventions and comparisons

All participating patients in the experimental group must receive rehabilitation nursing intervention. All participating patients in the comparison group can either receive no nursing intervention on any form of nursing, except rehabilitation nursing intervention.

#### 2.2.4 Types of outcome measures

The primary outcome includes health-related wellbeing. The secondary outcomes include psychological disorders (as per Beck Depression Inventory and Hamilton Depression Rating Scale), panic (measured by Panic Disorder Severity Scale), and adverse events.

### 2.3 Information sources and search strategy

#### 2.3.1 Electronic searches

The electronic databases listed below will be searched systematically: PubMed, EMBASE, CINAHL, Cochrane Library, Web of Science, Scopus, China National Knowledge Infrastructure, and WanFang database. All the databases will be searched from their inauguration to November 2020. There will be no language constraints. The keywords used in the search will be combined using “OR” or “AND” to identify studies related to: “burn∗,” “rehabilitation nursing,” “quality of life,” “RCT,” “randomized controlled trial,” “randomised controlled trial,” “prospective study.”

#### 2.3.2 Searching other sources

Furthermore, this study will also search other sources to prevent overlooking potential trials, such as, ongoing trials, conference proceedings, and reference lists of all primary studies.

### 2.4 Data collection and analysis

#### 2.4.1 Study selection

**Figure 1:**
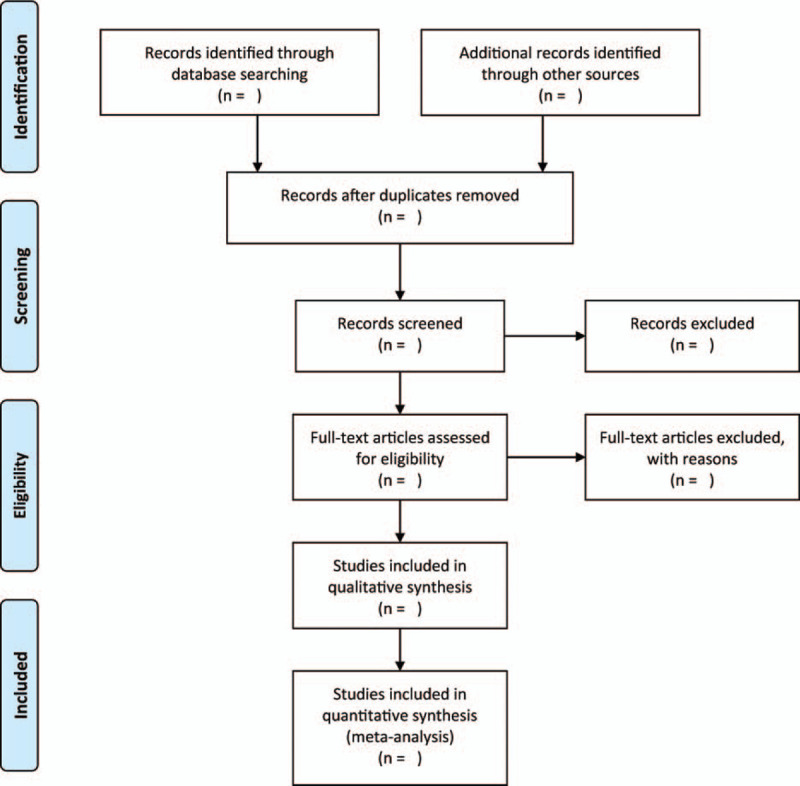
The research flowchart.

The titles and abstracts of the studies from the search will be scrutinized by 2 independent authors. Following this, the full-text will be scrutinized in detail to screen and select potential eligible studies. The present study includes all eligible studies. All disagreements will be resolved through discussions with a third author. Figure 1 shows a flowchart of the process of screening studies.

#### 2.4.2 Data extraction and management

The results from the systematic search will be imported into the EndNote X9 software, and duplicates will be removed. Based on the previously designed specialized datasheets, 2 authors will autonomously extract data from the selected studies. The following data will be extracted: first author, study design, study method, number of participants, age, gender, inclusion and exclusion criteria, interventions and comparisons details, and outcome indicators. All disagreements will be resolved by discussing with a third author.

#### 2.4.3 Dealing with missing data

The corresponding author of the study will be contacted through email or fax if data is incomplete or missing. In the cases of unavailable data, only available data will be included.

#### 2.4.4 Risk of bias assessment

Two autonomous authors will use the guidance of the Cochrane Risk of Bias Tool to assess the risk of bias of the RCTs study.^[11]^ Moreover, The Newcastle-Ottawa Scale will be adopted to assess the risk of non-RCTs.^[12]^ All disagreements will be resolved through discussion with a third author.

#### 2.4.5 Measures of treatment effect

For dichotomous data, this study will use the risk ratio combined with 95% confidence interval to estimate outcome results. For continuous data, this study will use the mean difference or standardized mean difference combined with 95% confidence intervals to determine the outcome results.

#### 2.4.6 Assessment of heterogeneity

Cochrane Q test and I2 statistic will be used to evaluate the statistical heterogeneity. An I2 > 50% or *P* < .1 implies substantial heterogeneity, in this case, the random-effects model will be used to integrate outcome data. Meanwhile, an *I*^2^ < 50% or *P* > .1 implies reasonable heterogeneity, consequently, this study will then use a fixed-effects model to combine outcome data.

#### 2.4.7 Sensitivity analysis

It is also planned to utilize a sensitivity analysis to assess the robustness and stability of the findings by sequentially removing low-quality studies.

#### 2.4.8 Assessment of reporting biases

In the case when the number of eligible studies exceed ten, it is planned to use Funnel plots and Egger regression to assess any potential publication bias.

### 2.5 Ethics and dissemination

Since this study does not use any patient data, it does not need an ethical approval.

## 3 Discussion

This study will present high-quality evidence pertaining to the evaluation of the impact of rehabilitation nursing intervention on the wellbeing of patients sustaining burn injuries. Additionally, it provides a reference for nurses and the development of clinical guidelines. To the best of the author's knowledge, this is the first study that will specifically focus on evaluating the impact of rehabilitation nursing intervention on the wellbeing of patients with burn injuries. An increasing number of studies have studied the impact of rehabilitation nursing intervention on the wellbeing of patients with burn injuries; however, the results lack conclusiveness. Therefore, this study will systematically investigate the clinical effects of rehabilitation nursing intervention on the wellbeing of patients with burn injuries. The work done in this study is equally important for both clinicians and patients.

## Author contributions

**Conceptualization:** Jun xiang.

**Data curation:** Jun xiang, qing yang, jing zhou.

**Formal analysis:** Jun xiang, qing yang, jing zhou, hong wu.

**Funding acquisition:** weiguo xie, weidong zhang.

**Investigation:** qing yang.

**Project administration:** weiguo xie, jing zhou, xiang gong, weidong zhang.

**Resources:** qing yang, xiang gong.

**Software:** Jun xiang, qing yang.

**Supervision:** jing zhou, xiang gong.

**Validation:** qing yang, weiguo xie, hong wu.

**Visualization:** qing yang, weiguo xie.

**Methodology:** jing zhou.

**Writing – original draft:** Jun xiang, hong wu.

**Writing – review & editing:** Jun xiang, weiguo xie, hong wu.
